# Translation and validation of the mindful eating behaviour scale in the Arabic language

**DOI:** 10.1186/s12888-023-04614-1

**Published:** 2023-02-23

**Authors:** Feten Fekih-Romdhane, Diana Malaeb, Mirna Fawaz, Nancy Chammas, Michel Soufia, Sahar Obeid, Souheil Hallit

**Affiliations:** 1 The Tunisian Center of Early Intervention in Psychosis, Department of Psychiatry “Ibn Omrane”, Razi Hospital, 2010 Manouba, Tunisia; 2 University, Faculty of Medicine of Tunis, Tunis El Manar , Tunis, Tunisia; 3 College of Pharmacy, Gulf Medical University, Ajman, United Arab Emirates; 4 School of Pharmacy, Lebanese International University, Beirut, Lebanon; 5 Faculty of Health Sciences, Beirut Arab University, Afeef Al Tiba, Tareek Al Jadida, Beirut, 1105 Lebanon; 6 School of Medicine and Medical Sciences, Holy Spirit University of Kaslik, P.O. Box 446, Jounieh, Lebanon; 7 Social and Education Sciences Department, School of Arts and Sciences, Lebanese American University, Jbeil, Lebanon; 8 Psychology Department, College of Humanities, Effat University, Jeddah, 21478 Saudi Arabia; 9 Applied Science Research Center, Applied Science Private University, Amman, Jordan; 10 Research Department, Psychiatric Hospital of the Cross, Jal Eddib, Lebanon

**Keywords:** Mindful eating, Mindful eating behaviour scale, Psychometric properties, Arabic

## Abstract

**Background:**

There has been a drastic increase in the prevalence of obesity and its related diseases in the Arabic-speaking countries during the last decades along with a lack of public awareness about this awareness about this public health problem. This calls for the development of novel prevention and intervention strategies that are based on new approaches, including mindful eating. In this context, we aimed through this study to explore the factor structure, composite reliability, measurement invariance across sex, convergent and divergent validity of an Arabic translation of the Mindful Eating Behaviour Scale (MEBS).

**Methods:**

A cross-sectional study carried out between September and November 2022, and enrolled 359 participants, all aged above 18 years old and recruited from all Lebanon governorates. The questionnaire used included socio-demographic questions, and the following scales: The Mindful Eating Behavior Scale (MEBS), Rosenberg Self-Esteem Scale (RSES), Intuitive Eating Scale-2, and Depression Anxiety Stress Scale (DASS-8).

**Results:**

McDonald’s ω values ranged from .82 to .95 or the four mindful eating domains, indicating the excellent internal consistency reliability of the scale. Our study also showed that fit indices from the confirmatory factor analysis confirmed the original four-factor structure model of the MEBS. Furthermore, our analyses suggested that configural, metric, and scalar invariance was supported across sex. Our results found no sex difference in all MEBS subscales scores. Finally, we found positive correlations between Focused eating, Hunger and satiety cues on one hand, and intuitive eating on the other hand. Moreover, greater Hunger and satiety cues scores were correlated with higher self-esteem and lower body mass index.

**Conclusion:**

Our findings support the psychometric reliability and validity of the Arabic MEBS. We suggest, accordingly, that the scale will be of high clinical and research utility, and will help in the development of information-based interventions focused on mindful eating that are aimed to combat eating disorders and obesity in the Arab world.

## Background

Obesity has increased steadily over the past decades, and has become a major public health concern worldwide [1]. Unhealthy eating behaviours and obesity are considered among the main health risks for various diseases [2], decrease in life expectancy [3] and reduced quality of life [4]. Commonly adopted approaches to prevent and treat obesity include improving food quality by increasing the proportion of consumed healthy food (fruit and vegetables), limiting calorie consumption, and encouraging physical activity (e.g., [5]). However, low adherence to these lifestyle strategies substantially limits their effectiveness [6], and may even lead to paradoxical weight gain [7]. Therefore, there is an obvious necessity to develop more effective weight loss interventions. One potentially successful avenue for altering unhealthy eating habits and promoting weight reduction is mindfulness-based interventions [8–10]; or more specifically, what has recently been called mindful eating behaviour [11].

Mindfulness is a present-focussed experience consisting of consciously and intentionally directing attention to the moment’s attributes [12], as well as maintaining a non-judgmental awareness of perceptions, feelings, and thoughts in the present moment [13]. Based on this principle, mindful eating behaviour is intended to help the individual to increase their awareness of signals relating to fullness and hunger [14, 15], therefore allowing them to lower their emotional response to eating [16, 17], appropriately respond to internal or external hunger cues [18, 19], and reduce food cravings induced by these cues [20, 21]. In addition, mindful eating behaviour enables the individual to adjust their attitudes toward food and better understand their food aversions and preferences [22]. As such, a growing evidence has documented positive effects of mindfulness-based interventions in reducing binge eating [23, 24], decreasing impulsive food choices and delaying eating onset [25, 26]. In addition, mindfulness has been found to significantly reduce uncontrolled and emotional eating [27], as well as body mass index (BMI) [28, 29]. Interestingly, mindfulness has also demonstrated positive effect on increasing fruit and vegetable consumption, decreasing fat and sugar consumption [30], and reducing motivations to eat palatable foods [31]; it has been linked to self-efficacy with regard to healthy eating [32–35].

At this point, it is important to note the cultural differences toward mindful eating and more specifically food attitude. Previous studies [36, 37] pointed out that subjects in individualistic societies tend to worry more about their weight and to adopt a negative attitude around food. In fact, it is an individual’s responsibility to eat correctly, mindfully and remain fit. If the individual fails, he or she is deemed irresponsible and is blamed. However, in collectivistic cultures, individuals belong to in-groups or collectives where members look after each other in exchange for loyalty [38]. Hence, collectivism is characterized by a pre-eminence of the family as the most important facet of everyday life and as the major source of emotional comfort and support [39]. One can infer that in collectivistic cultures, food may be regarded in a less mindful perception and a more positive way because it facilitates social interactions and enhances the benefits of others’ company [40].

Given these multiple, increasingly clear benefits of mindful eating behaviour, there has been considerable recent interest in developing instruments to assess this construct. Framson et al. [16] were the first to attempt developing an eating-specific mindfulness measure in 2009, i.e., the Mindful Eating Questionnaire (MEQ). It evaluates mindful eating through five dimensions: Disinhibition, Awareness, Emotional Response, External Cues, and Distraction [16]. However, some overlap between items has been identified, as well as a difficulty to assess mindful eating in general situations (MEQ items rather refer to very specific situations such as parties and restaurant). Later, a shortened version of the MEQ has been developed to address these limitations [41]. Overall, the MEQ in its two versions has been criticized by some researchers because of a lack of agreement with standard definitions and factor structure of mindfulness (e.g., [42]). For instance, the MEQ only focuses on the emotional and bodily experiences related to eating, and does not include a nonjudgement or acceptance aspects of mindful eating. To overcome these gaps, a more recent measure, i.e. the Mindful Eating Scale (MES), has developed in 2014 by Hulbert-Williams et al.; and is comprised of 28 items and six factors, i.e. acceptance, non-reactivity, awareness, act with awareness, unstructured eating and routine [43]. The MES has been developed with the aim of measuring the central features of mindfulness (non-judgement and attention) and to align with the existing general mindfulness scales. However, the MES still presented a number of flaws, such as the inclusion of items that do not seem to evaluate mindful eating (e.g., “I eat between meals” and “I snack when I’m bored”), or the inclusion of factors that measure the outcome of having learned skills of mindfulness rather than the mindful eating experience itself [44].

More recently, Winkens et al. developed a new scale, i.e. the Mindful Eating Behavior Scale (MEBS), aiming at assessing the attention component of mindful eating without involving emotional and external eating, and thus evaluate the independent effects of mindful eating [44]. The acceptance component has not been involved her because it has not been able to demonstrate any changes in eating behaviour [45]. The MEBS consists of 17 items and four domains: Focused Eating, Eating without Distraction, Eating with Awareness, and Hunger and Satiety Cues. The developers of the MEBS considered the following definition of mindful eating: Eating with awareness and attention; which has been mainly inspired by the following definition of mindfulness “An enhanced attention to and awareness of current experience or present reality” [46]. The MEBS has shown good psychometric properties in terms of internal consistency reliability and convergent validity in a large sample of Dutch adults [44]. Later, two more scales have been developed: the 30-item, seven-factor Mindful eating inventory (MEI) [47], and the 29-item, Four facet mindful eating scale (FFaMES) [48].

### The present validation study

In this study, our main goal was to provide a scale to measure the mindful eating behaviour for the Arabic-speaking population. To this end, we chose to translate and validate the MEBS in the Arabic language. We believe that this is relevant and highly needed, especially since there has been a drastic increase in the prevalence of obesity and its related diseases in the Arabic-speaking countries during the last decades [49]. Various causal factors have been identified, such as sociocultural-related barriers to physical activity practicing and greater consumption of unhealthy food. These alarming rates of obesity have raised major concerns among clinicians working in Arab settings, especially given the lack of public awareness about the interaction between obesity and chronic diseases [49]. As such, there is an obvious and urgent need to develop prevention and intervention strategies to combat obesity and unhealthy eating behaviours in the Arab context. One of the potential strategies is cultivating mindful eating behaviours. A first step toward developing such strategies is proving psychometrically sound scales to measure this construct that allows future experimental and intervention studies in the Arabic-speaking population. Apart from its psychometric characteristics and its theory-based factors allowing to exclusively assess the independent effects of mindful eating, we chose the MEBS for its brevity. Indeed, the MEBS enables to measure four domains of mindful eating using only 17 items, which would be more suitable in the low- to middle-income Arab countries, where longer scales may be challenging and costly to administer. Thus, we aimed through this study to explore the factor structure, composite reliability, measurement invariance across sex, convergent and divergent validity of an Arabic translation of the MEBS. We hypothesized that the Arabic MEBS will show an adequate internal reliability, an adequate fit of the data to a four-factor structure, invariance by sex, and good divergent validity as attested by its relationship with BMI, intuitive eating, self-esteem, and psychological distress.

## Methods

### Study design

A cross-sectional study was carried out between September and November 2022, and enrolled 359 participants through convenience sampling through several areas in Lebanon governorates. Participants received an online link to the survey. They were encouraged to visit the link which would guide them to the consent form, purpose of the study, anonymity, and the questionnaire. There were no fees for participating in the study. The link was shared among the participants and sent to all districts of Lebanon (Beirut, Mount Lebanon, North Lebanon, South Lebanon, and Bekaa) through social networks, using the snowball technique; the research team approached people they know, who were then asked to forward the link to the study to other family members or friends they know, who fulfil the inclusion criteria. All participants residing in Lebanon and above 18 years were eligible to participate and were asked to send the link to other subjects. Excluded were those who refused to fill out the questionnaire. Internet protocol (IP) addresses were examined to ensure that no participant took the survey more than once.

### Questionnaire

The questionnaire used was anonymous and in Arabic, the native language in Lebanon; it required approximately 20 min to complete. The questionnaire consisted of three parts. The first part of the questionnaire included an explanation of the study topic and objective, a statement ensuring the anonymity of respondents. The participant had to select the option stating *I consent to participate in this study* to be directed to the questionnaire.

The second part of the questionnaire contained sociodemographic information about the participants (age and sex). The Body Mass Index (BMI) was calculated using the self-reported weight and height [50].

The third part included the scales used in this study:

#### The MEBS [44]

This measure is made up of 17 items and four domains: (1) Focused Eating (five items; e.g., "I stay aware of my food while eating"), (2) Hunger and Satiety Cues (five items; e.g., "I trust my body to tell me to stop eating"), (3) Eating without Distraction (four items; e.g., "I think about things I need to do while I am eating"), and (4) Eating with Awareness (three items; e.g., "I snack without being aware that I am eating"). Due to low inter-factor correlations, the developers of the scale do not recommend a computation of a total score combining these four domains. Answer categories range from 1 (never) to 5 (very often). Higher scores refer to greater levels of mindful eating. It is of note that this scale was originally validated in people aged 55 years and older; however, the items are formulated in a way that can be applied to any age group. Besides, the MEBS has previously demonstrated good psychometric properties in samples of young adults (e.g., Spanish adults aged 36.9 ± 11.3 years [51], UK adults aged 23.13 ± 8.32 years [52] and 37.44 ± 12.33, UK undergraduate students aged 20.46 ± 3.25 [53]). Therefore, we decided to assess its psychometric properties among a sample of adults aged 18 years and above.

#### The rosenberg self-esteem scale (RSES)

Is used to evaluate trait self-esteem. It is composed of 10 items, in which 5 items are reversed. This scale is scored as a Likert scale, with a 4-point response from Strongly Disagree to Strongly Agree. Higher scores indicate higher self-esteem [54] (McDonald’s ω = 0.84 in this study).

#### Intuitive eating scale-2 [55]

Includes 23 items assessing four dimensions of intuitive eating: Eating for physical reasons rather than emotional reasons; unconditional permission to eat; reliance on hunger and satiety cues; and body-food choice congruence. Participants are asked to rate each item using a 5-point Likert scale, ranging from Strongly disagree (1) to Strongly agree (5), selecting the option that best describes their attitudes or behaviors. Higher scores indicate higher intuitive eating (McDonald’s ω = 0.90 in this study).

#### Depression anxiety stress scale (DASS-8)

The Arabic version of the DASS-8 comprises eight items, in three subscales: depression (three items e.g., felt down hearted and blue), anxiety (three items e.g., felt scared without reason), and stress (two items e.g., was using a lot of my mental energy); the total scores of the DASS-8 and its subscales range between 0 to 24, 0 to 9, 0 to 9, and 0 to 6, respectively [56] (McDonald’s ω = 0.91 in this study).

### Translation procedure

The forward and backward translation method was applied to different scales. The English version was translated to Arabic by a Lebanese translator who was completely unrelated to the study. Afterwards, a Lebanese psychologist with a full working proficiency in English, translated the Arabic version back to English. The initial English version and the second English version were compared to detect and later eliminate any inconsistencies.

### Statistical analysis

#### Confirmatory factor analysis

We used data from the total sample to conduct a CFA using the SPSS AMOS v.26 software. A previous study suggested that the minimum sample size to conduct a confirmatory factor analysis ranges from 3 to 20 times the number of the scale’s variables [57]. Therefore, we assumed a minimum sample of 250 participants needed to have enough statistical power based on a ratio of 15 participants per one item of the scale, which was exceeded in this sample. Parameter estimates were obtained using the robust maximum likelihood method and fit indices. Additionally, evidence of convergent validity was assessed in this subsample using the average variance extracted (AVE) value (≥ 0.50 considered adequate) [58].

#### Sex invariance

To examine sex invariance of the MEB, we conducted multi-group CFA [59] using the total sample as well. Measurement invariance was assessed at the configural, metric, and scalar levels [60]. Configural invariance implies that the latent scales’ variable(s) and the pattern of loadings of the latent variable(s) on indicators are similar across sexes (i.e., the unconstrained latent model should fit the data well in both groups). Metric invariance implies that the magnitude of the loadings is similar across sexes; this is tested by comparing two nested models consisting of a baseline model and an invariance model. Lastly, scalar invariance implies that both the item loadings and item intercepts are similar across sexes and is examined using the same nested-model comparison strategy as with metric invariance [59]. Following previous recommendations [59, 61], we accepted ΔCFI ≤ 0.010 and ΔRMSEA ≤ 0.015 or ΔSRMR ≤ 0.010 (0.030 for factorial invariance) as evidence of invariance. We aimed to test for sex differences on latent MEBS scores using an independent-samples *t*-test only if scalar or partial scalar invariance were established [62].

#### Further analyses

Composite reliability in both subsamples was assessed using McDonald’s (1970) ω and its associated 95% CI, with values greater than 0.70 reflecting adequate composite reliability [63]. McDonald’s ω was selected as a measure of composite reliability because of known problems with the use of Cronbach’s α (e.g., [64]. To assess convergent and criterion-related validity, we examined bivariate correlations between the MEB subscales scores and the additional measures included in the survey (DASS-8, RSES and IES). All scores had a normal distribution, as identified by skewness and kurtosis values varying between -1 and + 1 [65]; therefore, Pearson correlation test was used to correlate two continuous variables, whereas the Student t test was used for the comparison of two means. Based on [66], values ≤ 0.10 were considered weak, ~ 0.30 were considered moderate, and ~ 0.50 were considered strong correlations.

## Results

A total of 359 participants enrolled in this study (mean age: 22.75 ± 7.04 years, 40.1% males). Other sociodemographic characteristics are summarized in Table 1.

**Table 1:** Sociodemographic and other characteristics of the participants (*n* = 359)

Variable	n (%)
Sex	
Male	144 (40.1%)
Female	215 (59.9%)
	**Mean ± SD**
Age, years	22.75 ± 7.04
Body Mass Index (kg/m^2^)	24.12 ± 5.13
Intuitive eating	3.11 ± .36
Self-esteem	27.94 ± 4.19
Focused eating	17.10 ± 6.32
Hunger and satiety cues	15.71 ± 6.04
Eating with awareness	7.08 ± 3.34
Eating with distraction	10.17 ± 3.96

Intuitive eating = Score obtained from the Intuitive Eating Scale -2; Self-esteem = Score obtained from the Rosenberg Self-Esteem Scale; Focused eating, Hunger and satiety cues, Eating with awareness and Eating with distraction are the four domains deriving from the Mindful Eating Behavior Scale

### Confirmatory factor analysis

CFA indicated that fit of the four-factor model of the MEB scores was acceptable: χ^2^/df = 611.7/239 = 2.56, RMSEA = 0.066 (90% CI 0.060, 0.073), SRMR = 0.075, CFI = 0.934, TLI = 0.924. The standardised estimates of factor loadings were all adequate (Table 2). The convergent validity for this model was adequate, as AVE = 0.74.

**Table 2:** Items of the MEB in English and Standardized Estimates of Factor Loadings from the Confirmatory Factor Analysis (CFA) in the total sample

Item	CFA
Factor 1: Focused eating	
1	.85
2	.84
3	.94
4	.87
5	.87
Factor 2: Hunger and satiety cues	
6	.88
7	.93
8	.90
9	.78
10	.82
Factor 3: Eating with awareness	
12	.75
13	.92
14	.89
Factor 4: Eating with distraction	
15	.67
16	.77
17	.78
20	.69

### Composite reliability

Composite reliability of scores was adequate for the four factors in the total sample and in both males and females (Table 3).

**Table 3:** Composite reliability of the four factors items in the total sample and in both males and females

	Total sample	Males	Females
Focused eating	ω = .95	ω = .94	ω = .96
Hunger and satiety cues	ω = .95	ω = .93	ω = .96
Eating with awareness	ω = .87	ω = .89	ω = .86
Eating with distraction	ω = .82	ω = .82	ω = .83

### Sex invariance

As reported in Table 4, all indices suggested that configural, metric, and scalar invariance was supported across sex. The Student *t*-test results showed no sex difference in all MEBS subscales scores (Table 5).

**Table 4:** Measurement Invariance across sex in the total sample

Model	χ^2^	*df*	CFI	RMSEA	SRMR	Model Comparison	Δχ^2^	ΔCFI	ΔRMSEA	ΔSRMR	Δ*df*	*p*
Configural	854.87	326	.923	.067	.077							
Metric	867.85	342	.923	.066	.077	Configural vs metric	12.98	< .001	.001	< .001	16	.674
Scalar	966.39	360	.911	.069	.077	Metric vs scalar	98.54	.012	.003	< .001	18	< .001

*CFI* Comparative fit index, *RMSEA* Steiger-Lind root mean square error of approximation, *SRMR* Standardised root mean square residual

**Table 5:** Sex differences in terms of mindful eating behaviour subscales scores

	**Males**	**Females**	***p***	**Effect size**
Focused eating	17.72 ± 5.87	16.69 ± 6.59	.123	.165
Hunger and satiety cues	15.54 ± 5.67	15.82 ± 6.29	.671	.046
Eating with awareness	7.06 ± 3.40	7.09 ± 3.31	.927	.009
Eating with distraction	10.41 ± 4.02	10.01 ± 3.92	.349	.101

### Convergent and divergent validity

Higher focused eating scores were significantly associated with more intuitive eating, more psychological distress and older age. Higher hunger and satiety cues scores were significantly associated with more intuitive eating, higher self-esteem and lower BMI. Higher eating with awareness scores were significantly associated with more psychological distress. Finally, higher eating with distraction scores were significantly associated with lower self-esteem and higher psychological distress (Table 6).

**Table 6:** Correlation between mindful eating behaviors subscales scores and other continuous variables

	**1**	**2**	**3**	**4**	**5**	**6**	**7**	**8**	**9**
1. Focused eating	1								
2. Hunger and satiety cues	.72***	1							
3. Eating with awareness	.22***	.30***	1						
4. Eating with distraction	.36***	.44***	.68***	1					
5. Intuitive eating	.26***	.30***	-.09	.001	1				
6. Self-esteem	.10	.22***	-.06	-.11*	.29***	1			
7. Psychological distress	.19***	.09	.28***	.39***	.02	-.33***	1		
8. Age	.12*	.02	-.06	-.01	.04	.05	-.07	1	
9. BMI	.07	-.14**	.03	.04	-.12*	-.16**	-.01	.14**	1

Intuitive eating = Score obtained from the Intuitive Eating Scale -2; Self-esteem = Score obtained from the Rosenberg Self-Esteem Scale; Focused eating, Hunger and satiety cues, Eating with awareness and Eating with distraction are the four domains deriving from the Mindful Eating Behavior Scale; psychological distress = Score obtained from the Depression, Anxiety and Stress Scale- 8 items (DASS-8), *BMI* Body mass index

^*^p < .05

^**^p < .01

^***^p < .001

## Discussion

With the continued increase in the prevalence of obesity in Arab countries, it becomes urgent to develop new prevention and intervention approaches with novel targets that have proven efficacy in other contexts. In this paper, we propose to validate the Arabic version of the MEBS, with the aim of draw attention to this promising clinical and research avenue in the Arab settings. Our findings support the psychometric reliability and validity of the Arabic MEBS.

McDonald’s ω values ranged from 0.82 to 0.95 or the four mindful eating domains, indicating the excellent internal consistency reliability of the scale. These values further confirm the reliability of the MEBS that has been demonstrated in the original validation, where Cronbach’s alpha values varied between 0.717 and 0.907 for the four subscales [44]. Our study also showed that fit indices from the CFA confirmed the original four-factor structure model of the MEBS proposed by Winkens et al. [44]. Another study has also consistently replicated the same factor structure in an English-speaking population of British adults [67]. Furthermore, our analyses suggested that configural, metric, and scalar invariance was supported across sexes. This was in line with the original validation, in which model fit was satisfactory for sex groups in the Dutch adult sample [44]. This suggests that the Arabic MEBS seems to provide comparable measurements for individuals of both sexes. Our results found no sex difference in all MEBS subscales scores. In agreement with these findings, a Romanian study showed no influence of sex on mindful eating behaviour as assessing using the MEQ [68]. Similarly, a study among Turkish undergraduate students found no significant sex difference in MEQ scores [69].

As expected, we found positive correlations between Focused eating, Hunger and satiety cues on one hand, and intuitive eating on the other hand. Moreover, greater Hunger and satiety cues scores were correlated with higher self-esteem and lower BMI. These results support an adequate preliminary convergent validity of the Arabic MEBS. Intuitive eating refers to eating in response to innate satiety and hunger signals, without any restrictions on consumed food types [70]. It thus represents another approach to weight management that has been shown to influence food intake and quality, to the same extent as mindful eating [71]. In the original validation study, Winkens et al. has also documented positive correlations between three mindful eating domains of the MEBS (i.e., Focused Eating, Eating with Awareness, and Eating without Distraction) and self-esteem scores. Previous studies have also highlighted correlations in the same direction (e.g., [72]). Finally, consistent with our findings, the MEBS domains previously showed negative correlations with BMI [44]. However, it is also worth noting that prior research revealed mixed results on the link between mindful eating and BMI (for review, see [45]). Overall, our findings represent a preliminary but important attempt towards gaining a better knowledge of how eating mindfully relate to weight, intuitive eating, and mental health issues in the Arab social and cultural background.

### Limitations and research perspectives

This study has certain limitations that need to be addressed in future research. First, the cross-sectional design precludes drawing any causal conclusions. Second, due to the self-report nature of the questionnaire, the study may be subject to response bias. Third, even though our study targeted an Arabic-speaking population living in an Arab country (i.e., Lebanon), we are aware that further validation studies in other Arab contexts are still needed to ensure that the psychometric characteristics of our Arabic version of the MEBS are robust for replication in the broad Arabic-speaking community worldwide. Finally, even though we have been able to confirm measurement invariance for different sex groups, invariance across age, BMI and culture groups still needs to be demonstrated.

## Conclusion

In the present study, we provide an Arabic version of the 17-item MEBS, and confirm its psychometrically robust properties. We suggest, accordingly, that the scale will be of high clinical and research utility, and will help in the development of information-based interventions focused on mindful eating that are aimed to combat eating disorders and obesity in the Arab world.

## Data Availability

The datasets generated and/or analysed during the current study are not publicly available due to restrictions from the ethics committee but are available from the corresponding author on reasonable request.
